# Deciphering Evolutionary Dynamics and Lineage Plasticity in Aggressive Prostate Cancer

**DOI:** 10.3390/ijms222111645

**Published:** 2021-10-28

**Authors:** Natasha Kyprianou, Fabrice Lucien

**Affiliations:** 1 Department of Urology, Icahn School of Medicine at Mount Sinai, New York, NY 10029, USA; 2 Department of Urology, Mayo Clinic, Rochester, MN 55902, USA

This Special Issue focuses on the molecular mechanisms involved in therapeutic resistance, lineage plasticity, and phenotypic reprogramming leading to prostate cancer recurrence and, ultimately, lethal disease. Over the past decade, scientists and clinicians have teamed up to develop novel therapeutic agents for the treatment of advanced prostate cancer, such as second-generation androgen deprivation therapy, PARP inhibitors, and radionuclide therapy [1,2,3,4,5,6]. While these treatments led to improvements in patient overall survival, therapeutic resistance invariably develops, leading to mortality. Therefore, there is a critical need to understand the underpinnings of acquired resistance and develop effective therapeutic approaches for the treatment of advanced prostate cancer.

Androgen signaling is a major driver of prostate growth, prostate cancer development, and progression; thus, the blockade of the androgen receptor (AR)/androgen axis is effective in impairing tumor growth. Androgen deprivation therapy (ADT) and AR-targeting agents, particularly in combination with microtubule-targeting taxane chemotherapy, offer survival benefits in recurrent prostate cancer patients. However, most patients eventually develop castration-refractory disease, the most lethal form of prostate cancer. Understanding the mechanisms underlying resistance is critical to improving therapeutic outcomes. Recent advances in next-generation sequencing technologies have allowed for the characterization of the molecular landscape of metastatic prostate cancer leading to important insights on the mechanisms of therapeutic resistance and tumor progression to lethal disease [7,8,9,10,11]. High intratumoral heterogeneity and the presence of mixed phenotypes reveal evolutionary dynamics and the emergence of treatment-resistant populations which, ultimately, outcompete sensitive ones [12]. Some treatment-resistant clones emerged as a consequence of persistent androgen receptor addiction, which is reflected by the aberrant expression and amplification of the AR gene, de novo intraprostatic androgen production, and cross-talk between androgen signaling and other oncogenic pathways [13,14,15]. Other resistant clones become insensitive to androgens through the upregulation of constitutively active AR splice variants (i.e., AR-Vs) and the epigenetic reprogramming of AR activity [16,17,18]. In addition, genomic alterations on tumor suppressors RB1, TP53, and PTEN can contribute to the transition towards a resistant phenotype to antiandrogen therapy [19,20]. An emerging and significant subtype of treatment-resistant prostate cancer, called neuroendocrine (NEPC), is characterized by AR silencing, transcriptional reprogramming supporting proliferative capacity, and phenotypic switching towards stemness features [21,22,23,24,25]. No therapeutic strategy is available for the treatment of NEPC and patient outcome remains extremely poor.

Further work is needed to decipher the cascade of molecular and cellular events mediating lineage plasticity and the establishment of treatment-resistant tumor phenotypes. Emphasis should be given on the establishment of patient-derived xenograft models from metastatic biopsies and genetically engineered mouse prostate cancer models that recapitulate tumor evolution and intratumoral heterogeneity. Furthermore, a deep and comprehensive molecular profiling of metastatic tumors at different stages of the disease will be critical to understand the temporal and spatial determinants of treatment resistance. Molecular subtyping of metastatic castration-refractory prostate cancer will help treatment decision making and the identification of therapeutic vulnerabilities for drug discovery [9]. Given that a multiregional and longitudinal tumor biopsy can be very challenging for metastatic patients, it is anticipated that liquid biopsy (ctDNA, CTC, and extracellular vesicles) will become a preferred alternative to capture tumor heterogeneity and monitor lineage plasticity and treatment resistance in real time [26,27].

## References

[1] Fizazi K., Scher H.I., Molina A., Logothetis C.J., Chi K.N., Jones R.J., Staffurth J.N., North S., Vogelzang N.J., Saad F. (2012). Abiraterone acetate for treatment of metastatic castration-resistant prostate cancer: Final overall survival analysis of the COU-AA-301 randomised, double-blind, placebo-controlled phase 3 study. Lancet Oncol..

[2] Scher H.I., Fizazi K., Saad F., Taplin M.E., Sternberg C.N., Miller K., de Wit R., Mulders P., Chi K.N., Shore N.D. (2012). Increased survival with enzalutamide in prostate cancer after chemotherapy. N. Engl. J. Med..

[3] Parker C., Nilsson S., Heinrich D., Helle S.I., O’Sullivan J.M., Fossa S.D., Chodacki A., Wiechno P., Logue J., Seke M. (2013). Alpha emitter radium-223 and survival in metastatic prostate cancer. N. Engl. J. Med..

[4] Sartor O., de Bono J., Chi K.N., Fizazi K., Herrmann K., Rahbar K., Tagawa S.T., Nordquist L.T., Vaishampayan N., El-Haddad G. (2021). Lutetium-177-PSMA-617 for Metastatic Castration-Resistant Prostate Cancer. N. Engl. J. Med..

[5] Mateo J., Porta N., Bianchini D., McGovern U., Elliott T., Jones R., Syndikus I., Ralph C., Jain S., Varughese M. (2020). Olaparib in patients with metastatic castration-resistant prostate cancer with DNA repair gene aberrations (TOPARP-B): A multicentre, open-label, randomised, phase 2 trial. Lancet Oncol..

[6] Abida W., Patnaik A., Campbell D., Shapiro J., Bryce A.H., McDermott R., Sautois B., Vogelzang N.J., Bambury R.M., Voog E. (2020). Rucaparib in Men With Metastatic Castration-Resistant Prostate Cancer Harboring a BRCA1 or BRCA2 Gene Alteration. J. Clin. Oncol..

[7] Wang L., Dehm S.M., Hillman D.W., Sicotte H., Tan W., Gormley M., Bhargava V., Jimenez R., Xie F., Yin P. (2018). A prospective genome-wide study of prostate cancer metastases reveals association of wnt pathway activation and increased cell cycle proliferation with primary resistance to abiraterone acetate-prednisone. Ann. Oncol..

[8] Labrecque M.P., Coleman I.M., Brown L.G., True L.D., Kollath L., Lakely B., Nguyen H.M., Yang Y.C., da Costa R.M.G., Kaipainen A. (2019). Molecular profiling stratifies diverse phenotypes of treatment-refractory metastatic castration-resistant prostate cancer. J. Clin. Investig..

[9] Aggarwal R., Rydzewski N.R., Zhang L., Foye A., Kim W., Helzer K.T., Bakhtiar H., Chang S.L., Perry M.D., Gleave M. (2021). Prognosis Associated with Luminal and Basal Subtypes of Metastatic Prostate Cancer. JAMA Oncol..

[10] Quigley D.A., Dang H.X., Zhao S.G., Lloyd P., Aggarwal R., Alumkal J.J., Foye A., Kothari V., Perry M.D., Bailey A.M. (2018). Genomic Hallmarks and Structural Variation in Metastatic Prostate Cancer. Cell.

[11] Abida W., Cyrta J., Heller G., Prandi D., Armenia J., Coleman I., Cieslik M., Benelli M., Robinson D., Van Allen E.M. (2019). Genomic correlates of clinical outcome in advanced prostate cancer. Proc. Natl. Acad. Sci. USA.

[12] Zhang J., Cunningham J.J., Brown J.S., Gatenby R.A. (2017). Integrating evolutionary dynamics into treatment of metastatic castrate-resistant prostate cancer. Nat. Commun..

[13] Robinson D., Van Allen E.M., Wu Y.M., Schultz N., Lonigro R.J., Mosquera J.M., Montgomery B., Taplin M.E., Pritchard C.C., Attard G. (2015). Integrative clinical genomics of advanced prostate cancer. Cell.

[14] Locke J.A., Guns E.S., Lubik A.A., Adomat H.H., Hendy S.C., Wood C.A., Ettinger S.L., Gleave M.E., Nelson C.C. (2008). Androgen levels increase by intratumoral de novo steroidogenesis during progression of castration-resistant prostate cancer. Cancer Res..

[15] Chen C.D., Welsbie D.S., Tran C., Baek S.H., Chen R., Vessella R., Rosenfeld M.G., Sawyers C.L. (2004). Molecular determinants of resistance to antiandrogen therapy. Nat. Med..

[16] Antonarakis E.S., Lu C., Wang H., Luber B., Nakazawa M., Roeser J.C., Chen Y., Mohammad T.A., Chen Y., Fedor H.L. (2014). AR-V7 and resistance to enzalutamide and abiraterone in prostate cancer. N. Engl. J. Med..

[17] Li Y., Yang R., Henzler C.M., Ho Y., Passow C., Auch B., Carreira S., Nava Rodrigues D., Bertan C., Hwang T.H. (2020). Diverse AR Gene Rearrangements Mediate Resistance to Androgen Receptor Inhibitors in Metastatic Prostate Cancer. Clin. Cancer Res..

[18] Davies A., Nouruzi S., Ganguli D., Namekawa T., Thaper D., Linder S., Karaoglanoglu F., Omur M.E., Kim S., Kobelev M. (2021). An androgen receptor switch underlies lineage infidelity in treatment-resistant prostate cancer. Nat. Cell Biol..

[19] Ku S.Y., Rosario S., Wang Y., Mu P., Seshadri M., Goodrich Z.W., Goodrich M.M., Labbe D.P., Gomez E.C., Wang J. (2017). Rb1 and Trp53 cooperate to suppress prostate cancer lineage plasticity, metastasis, and antiandrogen resistance. Science.

[20] Mu P., Zhang Z., Benelli M., Karthaus W.R., Hoover E., Chen C.C., Wongvipat J., Ku S.Y., Gao D., Cao Z. (2017). SOX2 promotes lineage plasticity and antiandrogen resistance in TP53- and RB1-deficient prostate cancer. Science.

[21] Beltran H., Prandi D., Mosquera J.M., Benelli M., Puca L., Cyrta J., Marotz C., Giannopoulou E., Chakravarthi B.V., Varambally S. (2016). Divergent clonal evolution of castration-resistant neuroendocrine prostate cancer. Nat. Med..

[22] Yasumizu Y., Rajabi H., Jin C., Hata T., Pitroda S., Long M.D., Hagiwara M., Li W., Hu Q., Liu S. (2020). MUC1-C regulates lineage plasticity driving progression to neuroendocrine prostate cancer. Nat. Commun..

[23] Bishop J.L., Thaper D., Vahid S., Davies A., Ketola K., Kuruma H., Jama R., Nip K.M., Angeles A., Johnson F. (2017). The Master Neural Transcription Factor BRN2 Is an Androgen Receptor-Suppressed Driver of Neuroendocrine Differentiation in Prostate Cancer. Cancer Discov..

[24] Guo H., Ci X., Ahmed M., Hua J.T., Soares F., Lin D., Puca L., Vosoughi A., Xue H., Li E. (2019). ONECUT2 is a driver of neuroendocrine prostate cancer. Nat. Commun..

[25] Alumkal J.J., Sun D., Lu E., Beer T.M., Thomas G.V., Latour E., Aggarwal R., Cetnar J., Ryan C.J., Tabatabaei S. (2020). Transcriptional profiling identifies an androgen receptor activity-low, stemness program associated with enzalutamide resistance. Proc. Natl. Acad. Sci. USA.

[26] Beltran H., Romanel A., Conteduca V., Casiraghi N., Sigouros M., Franceschini G.M., Orlando F., Fedrizzi T., Ku S.Y., Dann E. (2020). Circulating tumor DNA profile recognizes transformation to castration-resistant neuroendocrine prostate cancer. J. Clin. Investig..

[27] Sperger J.M., Emamekhoo H., McKay R.R., Stahlfeld C.N., Singh A., Chen X.E., Kwak L., Gilsdorf C.S., Wolfe S.K., Wei X.X. (2021). Prospective Evaluation of Clinical Outcomes Using a Multiplex Liquid Biopsy Targeting Diverse Resistance Mechanisms in Metastatic Prostate Cancer. J. Clin. Oncol..

